# Immune reconstitution in ART treated, but not untreated HIV infection, is associated with abnormal beta cell function

**DOI:** 10.1371/journal.pone.0197080

**Published:** 2018-05-24

**Authors:** Emily K. Sims, Grace Park, Kieren J. Mather, Raghavendra G. Mirmira, Ziyue Liu, Samir K. Gupta

**Affiliations:** 1 Department of Pediatrics, Indiana University School of Medicine, Indianapolis, IN, United States of America; 2 Center for Diabetes and Metabolic Diseases, Indiana University School of Medicine, Indianapolis, IN, United States of America; 3 Herman B Wells Center for Pediatric Research, Indiana University School of Medicine, Indianapolis, IN, United States of America; 4 Department of Medicine, Indiana University School of Medicine, Indianapolis, IN, United States of America; 5 Biochemistry and Molecular Biology, Indiana University School of Medicine, Indianapolis, IN, United States of America; 6 Cellular and Integrative Physiology, Indiana University School of Medicine, Indianapolis, IN, United States of America; 7 Department of Biostatistics, Indiana University School of Medicine, Indianapolis, IN, United States of America; 8 Division of Infectious Diseases, Indiana University School of Medicine, Indianapolis, IN, United States of America; Rush University, UNITED STATES

## Abstract

HIV infection has been associated with increased diabetes risk, but prior work has mostly focused on insulin resistance, as opposed to beta cell effects, or included patients on antiretroviral therapies (ART) directly linked to metabolic toxicity. In this analysis, we measured markers of glucose homeostasis and beta cell function, stress, and death in fasting sera from a cross section of HIV+ individuals off ART (n = 43), HIV+ individuals on ART (n = 23), and HIV- controls (n = 39). Markers included glucose, HOMA%S, HOMA%B, proinsulin:C-peptide ratio (PI:C ratio), and circulating *preproinsulin (INS)* DNA. We performed multiple linear regressions with adjustments for age, sex, race, BMI, and smoking status. Compared to HIV- controls, HIV+ participants off ART exhibited similar beta cell function and insulin sensitivity, without increases in markers of beta cell stress or death. Specifically, in HIV+ participants with CD4 counts <350 cells/μL, PI:C ratios were lower than in HIV- controls (p<0.01), suggesting a reduction in intrinsic beta cell stress among this group. By contrast, HIV+ participants on ART had higher fasting glucose (p<0.0001) and lower HOMA%B (p<0.001) compared to HIV- controls. Among the entire HIV+ population, higher HIV RNA correlated with lower fasting glucose (r = -0.57, p<0.001), higher HOMA%B (r = 0.40, p = 0.001), and lower PI:C ratios (r = -0.42, p<0.001), whereas higher CD4 counts correlated with higher PI:C ratios (r = 0.2, p = 0.00499). Our results suggest that HIV seropositivity in the absence of ART does not worsen beta cell function or glucose homeostasis, but immune reconstitution with ART may be associated with worsened beta cell function.

## Introduction

The development and success of combination antiretroviral therapy (ART) has transformed the prognosis of individuals infected with Human Immunodeficiency Virus (HIV), effectively increasing lifespans to approaching those of HIV-uninfected individuals [1–3]. However, these successes have brought about a simultaneous evolution in the epidemiology of the HIV positive population, with an emergence of numerous medical comorbidities associated with chronic HIV infection [4]. Secondary complications, either due to the virus itself or related to toxicities from HIV therapies, can exacerbate these comorbidities and lead to significant negative effects on quality of life, as well as mortality [4]. Specifically, multiple studies have reported increased diabetes mellitus (DM) development among individuals infected with HIV compared to those without HIV infection [4–10].

Incident DM in HIV positive individuals seems to be most consistent with Type 2 Diabetes (T2D) in clinical presentation and underlying pathophysiology [11]. Similar to T2D, HIV infected patients developing diabetes typically present with a relative insulin deficiency associated with increased peripheral insulin resistance and may respond to treatment with oral diabetes medications [11]. Increased insulin resistance has been linked to chronic immune activation and inflammation associated with viral infection, HIV associated lipodystrophy, or independent effects of ART [12–14]. However, while T2D is also typified by beta cell dysfunction, the potential contribution of beta cell dysfunction to HIV-related DM remains much less characterized [15].

To address this knowledge gap, we endeavored to define the presence of abnormalities in beta cell health in nondiabetic HIV positive individuals, using biomarkers of whole body glucose homeostasis, intrinsic beta cell dysfunction, and beta cell death. We chose to compare these markers in 1) HIV positive individuals off ART stratified by a) relatively higher (HIV+/CD4≥350/μL) or b) lower CD4 counts ((HIV+/CD4<350/μL) 2) HIV positive individuals on ART with suppressed viremia (HIV+/ART+), and 3) HIV negative healthy controls (HIV-). To model whole body glucose metabolism we utilized the homeostatic model assessment (HOMA) to calculate HOMA%B and HOMA%S scores based on fasting serum C-peptide and glucose values [16]. In addition, to detect intrinsic beta cell stress or injury that may precede changes in fasting serum glucose or C-peptide values, we measured a circulating biomarker of beta cell stress (proinsulin:C-peptide ratio) and a biomarker of beta cell death (circulating unmethylated *preproinsulin* DNA). Both of these circulating biomarkers reflect the physiologic steps in insulin production and release, the hallmarks of a normally functioning beta cell [17–19]. mRNA transcribed from *preproinsulin* (*INS)* DNA is initially translated into preproinsulin, which undergoes further processing and modification in the beta cell ER and golgi to proinsulin and then mature insulin and C-peptide before release in secretory granules [18]. This process is impaired in stressed beta cells, such as those undergoing oxidative or inflammatory stress, leading to accumulation and extracellular release of proinsulin. Therefore, beta cell stress and dysfunction can be measured via elevations in the circulating proinsulin:C-peptide, or PI:C ratio [19]. Because beta cells contain proportionally large amounts of unmethylated *INS* DNA relative to other tissues, release of DNA by dying beta cells can be detected via measurements in circulating unmethylated *INS* DNA, while circulating methylated *INS* DNA can be increased in association with death and/or turnover of other cell types [17].

We predicted that differential measures of beta cell function or dysfunction among our participant groups may shed insight into contributions of beta cell dysfunction to HIV associated diabetes development. Our analysis did not reveal any increase in fasting glucose, HOMA%B scores, or biomarkers of beta cell stress or death among HIV+ participants off ART, but higher fasting glucose values and lower HOMA%B scores in HIV+/ART+ individuals. Interestingly, we actually detected a reduction in PI:C ratio, consistent with a reduction in intrinsic beta cell stress, specifically among HIV+ participants not on ART with a reduced CD4 count <350/μL. Our findings suggest that independent of complications from ART, HIV infection in isolation does not increase beta cell dysfunction, and higher CD4 cell counts are actually associated with worsened beta cell function.

## Material and methods

### Participants

Fasting banked serum samples were obtained from 66 adult HIV seropositive participants and 39 HIV seronegative participants who completed other local studies at Indiana University (ClinicalTrials.gov NCT00796822, NCT00864916, NCT00919724, NCT01270802, and NCT01962961) [20, 21]. Seropositive participants were recruited from the HIV outpatient clinics associated with the Indiana University Health medical system. Research was carried out according to the Code of Ethics of the World Medical Association (Declaration of Helsinki). The Indiana University institutional review board approved all studies. All participants, including both HIV+ and HIV- participants, were above 18 years of age and provided written, informed consent to have their sera samples stored and made available for future analysis. All samples studied were obtained at the baseline study visits, before any clinical interventions were performed. HIV positive individuals were grouped based on ART history and CD4 count, with 28 HIV+/CD4≥350 participants, 15 HIV+/CD4<350 and 23 HIV+/ART+ participants identified. HIV+ participants off ART had not received ART within 6 months of sample acquisition. HIV+/ART+ participants were ≥ 50 years old, had received ART for at least 6 months and were virologically suppressed (HIV-1 RNA level <75 copies/ml).

HIV- controls without medical comorbidities who were matched by age (± 10 years), sex, and smoking status (current vs. non-current) to the HIV+ off ART groups were also studied. Major exclusion criteria in all groups included known cardiovascular disease, a diagnoses of diabetes or fasting blood glucose >126 mg/dL, uncontrolled hypertension, thyroid abnormalities, systemic inflammatory disease other than hepatitis B or C coinfection, pregnancy or breast feeding during the study, creatinine clearance <50 ml/min, hemoglobin<9.0 g/dL, alanine or aspartate aminotransferase levels >3 times the upper limit of normal, total bilirubin >2.5 times the upper limit of normal, or ongoing fever or active infection/malignancy requiring treatment during the study visit. HIV+/ART+ individuals with a previous history of stavudine or didanosine (which have been previously associated with lipodystrophy and insulin resistance) for more than 7 days were excluded [22]. To avoid any sampling bias, all available banked samples from these groups meeting the above eligibility criteria were utilized.

### Assays

All assay measurements were performed in a blinded fashion. Measurements of HIV RNA levels (‘viral loads’), CD4 counts, and glucose were measured by the IU Health Clinical Pathology lab using standard assays. High sensitivity C reactive protein (hsCRP), interleukin 6 (IL-6), C-peptide, and total proinsulin were assayed using commercially available ELISAs [20, 21, 23, 24]. hsCRP was measured using a BNII Nephelometer (Siemens, Munich, Germany) with a minimum detection limit of 0.168 ng/mL with no upper bound and interassay CV of 1.56–5.43% [21]. The IL-6 chemiluminescent ELISA (R&D, Minneapolis, MN) has a detection range of 0.48 to 1500 pg/mL and interassay CV range of 3.69–12.48% [21]. The C-peptide ELISA (ALPCO, Salem, NH) has a recommended detection range of 20–3000 pmol/L and interassay CV range of 6.6–8.7% [24]. Total proinsulin was assayed using a chemiluminescent Stellux ELISA (ALPCO), which detects human intact proinsulin, human Des (31,32) proinsulin, and human Des (64,64) proinsulin [23]. Cross-reactivity with human C-peptide, human insulin, and lispro is <0.1%, <0.6%, and <0.2%, respectively. The proinsulin detection range is 5 pg/mL-3000pg/mL and interassay CV range is 6.0–9.7%. One HIV- control participant and one HIV+/ART+ participant had insufficient serum for proinsulin analysis; therefore, these individuals were excluded from group comparisons of PI:C ratios or analysis of correlations with proinsulin values. Serum levels of unmethylated and methylated preproinsulin (*INS*) DNA were quantified as previously described using a droplet digital PCR-based assay that detects differential methylation at the -69 bp site of the *INS* gene [25]. 10 samples (4 controls, and 6 HIV+ samples) with poor total DNA recovery (<10 ng/μL) were excluded from *INS* DNA analysis. As previously described, F2-isoprostane, a measure of oxidative stress, was assessed using an LC-MS/MS analytical method [21].

Beta cell function and insulin sensitivity were modeled using fasting glucose and C-peptide values in the Homeostasis Model Assessment 2 (HOMA2) calculator (https:///www.dtu.ox.ac.uk/homacalculator) [26]. Fasting PI:C ratios were calculated as molar ratios to obtain circulating proinsulin relative to C-peptide.

### Statistics

Our primary objective in this analysis was to compare differences in glucose homeostasis markers among our participant groups. Glucose homeostasis markers were log-transformed for further analysis to address right-skewness. Categorical variables were summarized by counts and percentages, and were compared across groups by Pearson chi-square tests. Continuous variables were summarized by means and standard deviations (SDs), except glucose homeostasis markers which were summarized by geometric means, and were compared across groups by analysis of variances (ANOVA) or Student’s t-tests as appropriate. Glucose homeostasis markers were further compared across groups with adjustment for the effects of age, sex, race, body mass index (BMI) and smoking status. In particular, multiple linear regressions were fitted to adjust for covariate effects (S1 Data), and least-square means were used to quantify the adjusted group means. Overall group differences were analyzed using ANOVA with additional comparisons of individual groups to HIV- controls. As a secondary exploratory analysis, to measure relationships with traditional immune parameters and identify new relationships with inflammatory markers, we calculated Pearson correlation coefficients to quantify associations between glucose homeostasis markers and HIV markers and serum inflammatory markers. This analysis was also performed with adjustment for ART use as a variable. No multiple comparison adjustments were performed to maximize detection of potentially biologically important relationships. Two-sided p-values ≤0.05 were considered statistically significant. All analyses were conducted using SAS 9.4 (SAS Institute, Cary, NC, USA).

## Results

### Participant characteristics

Participant population demographics are described in detail in Table 1. Mean age values were similar among HIV–controls and HIV+ participants off ART (HIV- controls: 34; HIV+/CD4≥350: 35; and HIV+/CD4<350: 37 years) due to intentional matching for these specific groups, but HIV+/ART+ participants were older (mean age of 53 years) (p<0.001 for overall ANOVA). Race was also significantly different among the groups (p = 0.036). We did not detect significant differences in the systemic markers of inflammation hsCRP and IL-6, or in the oxidative stress marker F2-isoprostanes between groups. The specific ART drug combinations used in the HIV+/ART+ group included tenofovir/ emtricitabine/ efavirenz (n = 10), tenofovir/ emtricitabine/ rilpivirine (n = 3), tenofovir/ emtricitabine/ darunavir/ritonavir (n = 3), tenofovir/ emtricitabine/ darunavir/ ritonavir/ raltegravir (n = 2), tenofovir/ emtricitabine/ atazanavir/ ritonavir (n = 1), tenofovir/ emtricitabine/ elvitegravir/ cobicistat (n = 1), tenofovir/ emtricitabine/ lopinavir/ ritonavir (n = 1), tenofovir/ emtricitabine/ dolutegravir/ rilpivirine (n = 1), and abacavir/ lamivudine/ dolutegravir (n = 1).

**Table 1:** Group demographics.

Parameter	HIV–Controls (n = 39)	HIV+/ CD4≥350 cells/μL (n = 28)	HIV+/ CD4<350 cells/μL (n = 15)	HIV+/ART+ (n = 23)
Age (years)***	34(10)	35 (11)	37 (12)	53 (3)
**Gender (% Male)**	76.92	78.57	73.33	86.96
**Race (% nonBlack)**	66.67	42.86	26.67	43.38
**BMI (kg/m**^**2**^**)**	27.72 (7.12)	26.71 (4.97)	27.27 (7.04)	28.93 (5.14)
**Current Smokers (%)**	19 (48.72)	14 (50.00)	7 (46.67)	6 (26.09)
**CD4 Count (cells/ μL)**	887.76 (321.46)	556.71 (149.63)	138.93 (109.64)	661.00 (200.83)
**HIV RNA (log copies/ml)**	n/a	9.64(2.24)	11.03 (2.24)	3.16 (0.33)
**hsCRP (μg/ml)**	2.53 (3.03)	2.05 (2.07)	5.20 (11.28)	3.78 (2.90)
**IL-6 (pg/ml)**	3.28 (9.50)	2.55 (2.54)	3.27 (2.46)	2.77 (2.42)
**F2-isoprostanes (pg/mL)**	30.16 (15.6)	28.42 (14.88)	20.40 (9.52)	31.19 (25.8)

Results for continuous variables are displayed as mean (standard deviation) and categorical variables are displayed as frequency (percentage). HIV RNA data were log transformed.

*p<0.05

**p<0.01

***p<0.001 for analysis of overall differences in means among the four groups.

### Markers of glucose homeostasis and beta cell health

To define differences in traditional measures of glucose homeostasis among our participant groups, we compared fasting glucose as well as HOMA%S and HOMA%B values, respectively, as estimators of glucose tolerance, insulin sensitivity, and beta cell function [26]. Comparisons of geometric means, unadjusted for demographic variables, are shown in Table 2. Because significant demographic differences existed between our study groups, we also performed a multivariable regression analysis which adjusted for effects of age, race, sex, BMI, and smoking status. Geometric means from this analysis are presented in Table 3. Interestingly, there were no differences in glucose or HOMA scores, before or after adjustment, between HIV+ groups off ART and HIV- controls. By contrast, compared to HIV- controls, HIV+/ART+ participants had higher fasting glucose (HIV- controls: mean value of 81.12 mg/dL vs. HIV+/ART+: 103.79 mg/dL; p<0.001), and lower HOMA%B (HIV- controls: 114.72 vs. HIV+/ART+: 81.10; p<0.01). Adjustment for demographics and BMI did not impact these relationships (HIV- controls: mean fasting glucose 81.88 mg/dL vs. HIV+/ART+: 98.77 mg/dL; p<0.001, and HIV- controls: mean HOMA%B: 115.42 vs. HIV+/ART+: 77.77; p<0.001). Comparisons between the HIV+ groups off and on ART suggest that glucose tolerance and beta cell insulin secretion, as estimated by traditional parameters, are not impaired in individuals with untreated HIV infection. Rather, changes in fasting glucose and HOMA%B are linked to treatment with ART. None of the HIV+ groups exhibited significant reductions in HOMA%S, pointing away from a significant effect of HIV seropositivity or ART treatment on insulin sensitivity.

**Table 2:** Unadjusted values for markers of glucose homeostasis.

Parameter	HIV–Controls (n = 39)	HIV+/ CD4≥350 cells/μL (n = 28)	HIV+/ CD4<350 cells/μL (n = 15)	HIV+/ ART+ (n = 23)	Overall P Value
**Fasting Glucose (mg/dL)**	81.12	83.69	81.70	103.79***	<0.0001
**HOMA % S**	107.83	108.76	99.34	83.13	0.1846
**HOMA % B**	114.72	105.54	118.66	81.10**	0.0017
**PI:C Ratio**	0.0228	0.0223	0.0147**	0.0231	0.0435
**Unmethylated *INS* DNA (copies/μL)**	1.14	0.80	0.38*	1.08	0.2258
**Methylated *INS* DNA (copies/μL)**	4.56	2.67*	2.03**	7.30	0.0001

Results are displayed as geometric mean (exponential of the mean of the log-transformed variable).

*p<0.05

**p<0.01

***p<0.001 compared to HIV negative controls

**Table 3:** Values for markers of glucose homeostasis adjusted for age, race, sex, BMI, and smoking status.

Parameter	HIV–Controls (n = 39)	HIV+/ CD4≥350 cells/μL (n = 28)	HIV+/ CD4<350 cells/μL (n = 15)	HIV+/ ART+ (n = 23)	Overall P Value
**Fasting Glucose (mg/dL)**	81.88	84.12	81.55	98.77***	0.0003
**HOMA % S**	104.15	100.77	93.63	100.81	0.8768
**HOMA % B**	115.42	110.17	124.20	77.77***	0.0008
**PI:C Ratio**	0.0221	0.0215	0.0138**	0.0210	0.0333
**Unmethylated *INS* DNA (copies/μL)**	1.11	0.83	0.41	1.07	0.0458
**Methylated *INS* DNA (copies/μL)**	3.44	2.19	1.77	7.94*	0.0004

Results are displayed as geometric mean (exponential of the mean of the log-transformed variable).

*p<0.05

**p<0.01

***p<0.001 compared to HIV negative controls

As traditional measures of glucose homeostasis were not impacted by HIV infection itself, we then asked whether more sensitive biomarkers of beta cell stress and death may be altered and ultimately predispose HIV infected individuals to diabetes development. To address this question, we measured fasting PI:C ratios and circulating *INS* DNA as biomarkers of beta cell stress and destruction, respectively. Interestingly, direct comparisons of the HIV+/CD4≥350 or HIV+/ART+ groups to HIV- controls showed no difference in PI:C ratio. However, in both our unadjusted and adjusted analyses, PI:C ratios were lower in the HIV+/CD4 <350 participants (for unadjusted analysis, HIV- controls mean value: 0.0228 vs. HIV+/CD4 <350: 0.0147, p<0.01; for multivariate analysis, HIV- controls: 0.0221 vs. HIV+/CD4 <350: 0.0138, p<0.01 for HIV- controls vs. HIV+/CD4 <350).

Similar to PI:C ratios, concentrations of unmethylated *INS* DNA, a biomarker of beta cell death, were also significantly lower in the HIV+/CD4 <350 group compared to controls (HIV- controls: 1.14 copies/μL vs. HIV+/CD4 <350: mean value: 0.38 copies/μL p = 0.0435). However, this relationship was no longer significant after adjustment for demographics. No differences in unmethylated *INS* DNA were detected between either the HIV+/CD4≥350 or HIV+/ART+ groups compared to HIV negative controls. We also measured circulating methylated *INS* DNA, which although not beta cell specific, may be linked to non-specific systemic inflammation [27]. In our unadjusted analyses, methylated *INS* DNA was significantly lower in HIV+/ CD4≥350 and HIV+/CD4<350 participants compared to HIV- controls (For unadjusted analysis, HIV- controls: 4.56 copies/μL vs. HIV+/ CD4≥350 mean levels: 2.67 copies/μL and HIV+/ CD4<350 mean levels: 2.03 copies/μL; p<0.05 and p<0.01 for HIV+ groups vs. HIV-controls, respectively. For multivariable analysis, these trends were no longer significant, while values in the HIV+/ART+ group were actually higher than in HIV-controls: HIV- controls: 3.44 copies/μL vs. HIV+/ART+ mean levels: 7.94 copies/μL; p<0.05).

### Correlations with immune parameters

Next, to better understand the etiologies of the observed differences among our participant groups, we examined correlations between our measured markers of glucose homeostasis and beta cell stress/dysfunction and immune parameters measured in HIV infection. Here we looked at associations among the entire population (Table 4), as well as associations adjusted for ART use (Table 5). Interestingly, higher HIV RNA levels were negatively correlated with fasting glucose, PI:C ratios, and methylated *INS* DNA levels, and had a positive correlation with HOMA%B scores (Table 4). After adjustment for ART use, only relationships with PI:C ratio (r = -0.56, p<0.001) and methylated *INS* DNA (r = 0.26, p = 0.04) were significant (Table 5).

**Table 4:** Correlations between glucose homeostasis markers, HIV markers, and serum inflammatory markers among entire study population.

	Fasting glucose	HOMA %B	HOMA %S	PI:C Ratio	Unmethylated *INS* DNA	Methylated *INS* DNA
**Log (HIV1-RNA)**	**-0.57 (< .0001)**	**0.40 (0.001**	0.11 (0.41)	**-0.42 (0.0004)**	-0.08 (0.55)	**-0.32 (0.0095)**
**CD4 count**	-0.06 (0.57)	0.06 (0.52)	-0.01 (0.96)	**0.20 (0.0499)**	**0.29 (0.0036)**	**0.30 (0.0022)**
**hsCRP**	0.08 (0.44)	0.08 (0.45)	-0.16 (0.11)	0.15 (0.14)	-0.03(0.80)	0.11 (0.30)
**IL-6**	-0.01 (0.93)	0.04 (0.67)	-0.04 (0.68)	**0.23 (0.0217)**	-0.07 (0.49)	-0.05 (0.59)
**F2-isoprostanes**	0.06 (0.54)	-0.07 (0.47)	0.02 (0.86)	0.16 (0.11)	0.17 (0.09)	0.12 (0.24)

Results are displayed as r (p value); n = 99–102 except for HIV RNA level, where n = 64–65; statistically significant results are bolded.

**Table 5:** Correlations between glucose homeostasis markers, HIV markers, and serum inflammatory markers among entire study population, adjusted for history of ART.

	Fasting glucose	HOMA %B	HOMA %S	PI:C Ratio	Unmethylated *INS* DNA	Methylated *INS* DNA
**Log (HIV1-RNA)**	0.11 (0.39)	0.12 (0.34)	-0.22 (0.08)	**-0.56 (< .0001)**	-0.02 (0.90)	**0.26 (0.04)**
**CD4 count**	-0.11 (0.29)	0.09 (0.34)	0.01 (0.96)	**0.19 (0.05)**	**0.29 (0.0036)**	**0.30 (0.0022)**
**hsCRP**	0.04 (0.69)	0.12(0.26)	-0.15 (0.14)	0.14 (0.15)	-0.02 (0.82)	0.09 (0.40)
**IL-6**	0.01 (0.99)	0.04 (0.69)	-0.05 (0.66)	**0.23 (0.02)**	-0.07 (0.49)	-0.05 (0.61)
**F2-isoprostanes**	0.02 (0.823)	-0.05 (0.62)	0.03 (0.73)	0.15 (0.12)	0.17(0.09)	0.10 (0.33)

Results are displayed as r (p value); n = 99–102 except for HIV RNA level, where n = 64–65; statistically significant results are bolded.

Among all participants, CD4 cell count was not correlated with glucose or either HOMA score, but positively correlated with PI:C ratio, and both unmethylated and methylated *INS* DNA concentrations, suggesting that higher CD4 concentrations were linked to increases in measured markers of intrinsic beta cell stress and death (Table 4). Importantly, these relationships persisted after adjustment for treatment with ART as a variable (for CD4 and PI:C: r = 0.19, p = 0.05; for CD4 and unmethylated *INS* DNA: r = 0.29, p = 0.0036; for CD4 and methylated INS DNA: r = 0.30, p = 0.0022), suggesting a true correlation between higher CD4 counts and increased markers of beta cell stress and death, and pointing away from clustering of high CD4 counts in the ART group as the reason for this observed relationship. The only study subgroup where there was a significant correlation between CD4 counts and PI:C ratios was the HIV+/CD4<350 subgroup (r = 0.72, p = 0.0026).

We also analyzed correlations with the systemic inflammatory biomarkers hsCRP and IL-6, which have previously been linked to incident diabetes in HIV positive individuals [5, 28]. No correlations of metabolic markers with hsCRP were present, before or after adjustment for ART. Higher IL6 levels correlated with higher PI:C ratios, before and after adjustment for ART (before adjustment: r = 0.23, p = 0.0217; after adjustment: r = 0.23, p = 0.02). To identify any relationships with oxidative stress, we also measured circulating F2-isoprostanes. No significant correlations between F2-isoprostanes and beta cell biomarkers were identified.

## Discussion

Associations between HIV seropositivity and diabetes have been previously described, although the innate contribution of HIV infection on glucose homeostasis is not clear. Previous studies on this topic are complicated by inclusion of individuals receiving ART regimens containing the thymidine analogs stavudine and didanosine and first-generation protease inhibitors, which have been independently implicated in lipodystrophy and development of diabetes in HIV, or have focused on insulin resistance rather than beta cell function. In the current work, we analyzed a cross-section of serum samples from HIV+ individuals that were not receiving HIV-related therapy, and individuals that had achieved viral suppression on more modern and less metabolically active ART regimens. Our HIV- controls were carefully matched to the HIV+ participants not on ART. Furthermore, in addition to glucose and a marker of insulin sensitivity (HOMA%S), we specifically assessed several markers of beta cell function and health, including HOMA%B, PI:C ratios, and circulating *INS* DNA, to test effects of HIV infection on the beta cell. Our results point away from HIV infection itself as a direct contributor to beta cell death or dysfunction. Surprisingly, our findings actually suggest that beta cell stress may be slightly decreased in HIV+ individuals with low CD4 counts, with worsened beta cell function in those receiving ART.

The underlying etiology of diabetes associated with HIV infection has likely evolved with the progress of treatment options for HIV. Several classes of ART, including protease inhibitors (PIs) and nucleoside analogue reverse transcriptase inhibitors (NRTIs), have been associated with insulin resistance and diabetes development, with increasing incidence of diabetes associated with higher cumulative doses of combination ART [7, 22, 29–35]. Some of these effects have been linked to ART-related lipodystrophy [36, 37]. Importantly we excluded patients who had previously received stavudine or didanosine, as these drugs have been associated with insulin resistance and lipodystrophy [22]. Additionally, direct effects of ART on glucose uptake, insulin release (e.g. protease inhibitors), and mitochondrial function (e.g. thymidine analog nucleoside reverse transcriptase inhibitors) have been described [7, 22, 34, 38–41]. To exclude a specific effect of protease inhibitor treatment on insulin release as an explanation for our findings, we examined markers of beta cell function in the 8 individuals on protease inhibitors and found they were not significantly different from the HIV+ART+ group overall (data not shown). The relationship between initial ART agents and metabolic disturbances is supported by epidemiologic data showing diabetes incidence in the HIV positive population has modestly decreased with the introduction of newer, less metabolically toxic ART agents [5, 22, 42]. However, our data suggest that there may still be a potentially higher risk of incident diabetes in those receiving more modern ART as our HIV+ART+ study group had elevations in fasting glucose and reductions in HOMA%B compared to controls. Of note, we did not detect increases in PI:C ratios or unmethylated *INS* DNA in this group. This could be due to the cross-sectional nature of our data. Alternatively, these findings may reflect a direct effect on beta cell insulin secretion as responsible for our observed changes in glucose and HOMA%B. Analysis of longitudinal samples in the same individuals would provide further insight into these differences.

Certain conventional risk factors for diabetes development are likely occurring in the HIV positive population. A large scale analysis of over 30,000 HIV infected veterans identified a significant interaction of age and HIV positivity on diabetes risk [43]. Several analyses have detected associations between traditional factors like age, BMI, race, waist circumference, and waist:hip ratio and diabetes development, even more than specific ART exposures [5, 6, 22, 42, 44]. Higher BMIs have been associated with progressive risk of incident diabetes, independent of medication history [5, 32]. Direct comparison of obese HIV positive individuals on treatment to obese HIV negative individuals demonstrated no significant differences in glucose or insulin resistance but did suggest differences in lipids and endothelial adhesion markers [45]. However, the impact of traditional risk factors on diabetes development does appear to be more pronounced based on studies evaluating benefits of traditional treatment approaches to Type 2 Diabetes that have reported blunted responses in HIV seropositive patients [30, 46, 47]. These differences may also be related to confounding factors among the HIV positive population and require further investigation [48].

Case reports have also suggested that HIV infection itself may be a risk factor for diabetes, with new diagnoses of HIV infection concurrently presenting with new onset diabetes that resolved after starting ART [49, 50]. Most commonly, HIV seropositivity is linked to insulin resistance [51, 52]. Biochemical markers of insulin resistance have been reported to be increased in HIV+ men compared to seronegative controls, regardless of treatment status [53]. Potential etiologies of such an effect could be systemic inflammation associated with viral infection or a HIV-induced proinflammatory shift in lipid profiles. Along these lines, incident diabetes in HIV+ individuals on ART has been associated with increased levels of circulating inflammatory markers, independent of BMI, including hsCRP, IL-6, and TNFR1 and TNFR2 levels [5, 28]. Alternatively, direct effects of HIV virus proteins on processes affecting insulin sensitivity have been identified, including increased glucocorticoid sensitivity, inhibition of adipocyte peroxisome proliferator-activated receptor γ (PPARγ) activity, and inhibition of adipocyte glucose transporter 4 (GLUT4) trafficking) [54–56].

Our results suggest that HIV infection alone may not lead to abnormalities in glucose homeostasis or markers of beta cell stress or death. By contrast, higher HIV RNA levels were actually associated with lower fasting glucose values, lower PI:C ratios, and higher HOMA%B scores, suggesting better beta cell function. We also observed reductions in PI:C ratios in seropositive participants with reduced CD4 counts, even after correction for confounding demographic factors. Interestingly, higher CD4 counts were positively correlated with PI:C ratios. Correlations between HIV RNA and CD4 counts and PI:C ratio, a marker of beta cell stress, were also present after adjustment for ART, suggesting that these observations were not merely a reflection of reduced HIV RNA and increased CD4 levels in the HIV+/ART+ group. The finding that higher CD4 counts are associated with markers of beta cell dysfunction could reflect a negative beta cell effect of increasing CD4 counts in the context of HIV. Interestingly, there is some precedent for a protective effect of immunosuppression in this context, as immune reconstitution and restoration of CD4 counts in immunosuppressed HIV positive individuals is associated with significant worsening of HIV related bone loss [57, 58].

Because we were interested in beta cell effects of HIV infection, and hyperglycemia-related glucose toxicity can induce beta cell dysfunction, we chose to exclude individuals who had already developed diabetes from this analysis [59]. However, it is possible that by failing to analyze those who already had diabetes, we underestimated the potential risks of HIV infection to affect beta cell function. Additionally, HOMA scores only provide estimates of beta cell function and insulin sensitivity that must be interpreted with caution [26]. Similarly, although both PI:C ratios and *INS* DNA are elevated in the context of both type 1 and T2D, these markers are not direct measurements of beta cell function or death [19, 60–64]. Nonetheless, in the absence of more invasive, costly, and time consuming measures, these markers can provide valuable information regarding glucose homeostasis [26].

In an effort to identify potentially biologically relevant associations, our correlation analysis did not adjust for multiple comparisons. Thus, our correlation analysis must be considered as exploratory. Larger scale prospective, longitudinal studies are indicated to more directly determine relationships detected between CD4 counts, HIV RNA and beta cell function. Another limitation of our study is the relatively higher number of male participants, which may not provide a representative picture of females who are seropositive for HIV. It has been reported that outcomes of HIV infection and ART can be sex dependent, and HIV-positive female patients have increased general immune activation and inflammatory reactions [65, 66]. Interestingly, comparison of HIV positive men and women suggests that female sex is associated with improved insulin sensitivity, although this effect is most likely independent of HIV positive status [67]. Along these lines, incidence of diabetes in HIV infected women has been reported at similar rates as uninfected women [29]. Further investigation into the pathogenesis of HIV-associated diabetes in women is indicated to shed light on sex related differences.

Notwithstanding these limitations, our findings suggest that HIV infection independent of ART does not substantially increase beta cell dysfunction, beta cell death, or whole-body glucose dyshomeostasis, while treatment with ART and higher CD4 counts were associated with beta cell dysfunction. Future efforts are indicated to better understand pathophysiologic contributors to diabetes development in HIV positive individuals and optimize therapy for HIV associated diabetes.

## Supporting information

S1 Data Supplemental data file-regression results. Results for multiple linear regression analyses fitted to adjust for covariate effects among each subject group, performed using SAS 9.4.
(DOCX)
