# Supplementary material for: Immune reconstitution in ART treated, but not untreated HIV infection, is associated with abnormal beta cell function
Source: PLoS One. 2018 May 24;13(5):e0197080. doi: 10.1371/journal.pone.0197080 (PMC5967701; doi:10.1371/journal.pone.0197080)
Supplement: S1 Data — Results for multiple linear regression analyses fitted to adjust for covariate effects among each subject group, performed using SAS 9.4. (DOCX) [file pone.0197080.s001.docx]

Regression results.

1. Log(glucose) as outcome.

| **Parameter** | **Estimate** | **Standard Error** | **P value** |
| --- | --- | --- | --- |
| **Intercept** | 4.269592192 | 0.08572512 | <.0001 |
| **Group: HIV+CD4>350 vs HIV-** | 0.026907022 | 0.03343867 | 0.4230 |
| **Group: HIV+CD4<350 vs HIV-** | -0.004096368 | 0.04148474 | 0.9216 |
| **Group: HIV+ART+ vs HIV-** | 0.187488361 | 0.04453401 | <.0001 |
| **Age** | 0.001757641 | 0.00138451 | 0.2074 |
| **Black versus non-black** | -0.018125134 | 0.02883890 | 0.5312 |
| **Female versus male** | -0.013805374 | 0.03507470 | 0.6948 |
| **BMI** | 0.003504730 | 0.00232932 | 0.1358 |
| **Current smoker** | -0.031376141 | 0.02688870 | 0.2462 |

1. Log(HOMA % S) as outcome

| **Parameter** | **Estimate** | **Standard Error** | **P value** |
| --- | --- | --- | --- |
| **Intercept** | 6.378286719 | 0.26690214 | <.0001 |
| **Group: HIV+CD4>350 vs HIV-** | -0.033012627 | 0.10411011 | 0.7519 |
| **Group: HIV+CD4<350 vs HIV-** | -0.106505329 | 0.12916128 | 0.4117 |
| **Group: HIV+ART+ vs HIV-** | -0.032527559 | 0.13865507 | 0.8150 |
| **Age** | -0.010950189 | 0.00431062 | 0.0127 |
| **Black versus non-black** | -0.093760883 | 0.08978890 | 0.2991 |
| **Female versus male** | -0.003547736 | 0.10920383 | 0.9742 |
| **BMI** | -0.044863302 | 0.00725227 | <.0001 |
| **Current smoker** | -0.038534642 | 0.08371703 | 0.6464 |

1. Log(HOMA % B) as outcome

| **Parameter** | **Estimate** | **Standard Error** | **P value** |
| --- | --- | --- | --- |
| **Intercept** | 3.850679292 | 0.21877421 | <.0001 |
| **Group: HIV+CD4>350 vs HIV-** | -0.046584164 | 0.08533692 | 0.5864 |
| **Group: HIV+CD4<350 vs HIV-** | 0.073318043 | 0.10587085 | 0.4903 |
| **Group: HIV+ART+ vs HIV-** | -0.394777548 | 0.11365272 | 0.0008 |
| **Age** | 0.004001322 | 0.00353333 | 0.2603 |
| **Black versus non-black** | 0.102413107 | 0.07359812 | 0.1674 |
| **Female versus male** | 0.035710572 | 0.08951214 | 0.6908 |
| **BMI** | 0.022835867 | 0.00594454 | 0.0002 |
| **Current smoker** | 0.095184937 | 0.06862113 | 0.1687 |

1. Log(PI:C ratio)

| **Parameter** | **Estimate** | **Standard Error** | **P value** |
| --- | --- | --- | --- |
| **Intercept** | 0.5381652674 | 0.34336719 | 0.1204 |
| **Group: HIV+CD4>350 vs HIV-** | -.0279323723 | 0.13367992 | 0.8349 |
| **Group: HIV+CD4<350 vs HIV-** | -.4702761301 | 0.16719450 | 0.0060 |
| **Group: HIV+ART+ vs HIV-** | -.0506468961 | 0.17917061 | 0.7780 |
| **Age** | -.0040401063 | 0.00553952 | 0.4676 |
| **Black versus non-black** | -.1435620646 | 0.11573664 | 0.2179 |
| **Female versus male** | -.1453809629 | 0.14108956 | 0.3054 |
| **BMI** | 0.0224294867 | 0.00938142 | 0.0188 |
| **Current smoker** | -.1443629503 | 0.10811450 | 0.1850 |

1. Log(unmethylated INS DNA) as outcome

| **Parameter** | **Estimate** | **Standard Error** | **P value** |
| --- | --- | --- | --- |
| **Intercept** | -2.034507512 | 0.86642845 | 0.0209 |
| **Group: HIV+CD4>350 vs HIV-** | -0.167172199 | 0.33731845 | 0.6213 |
| **Group: HIV+CD4<350 vs HIV-** | -0.473475724 | 0.42188677 | 0.2646 |
| **Group: HIV+ART+ vs HIV-** | -0.199442295 | 0.45210643 | 0.6601 |
| **Age** | 0.008172489 | 0.01397803 | 0.5602 |
| **Black versus non-black** | 0.939333014 | 0.29204165 | 0.0018 |
| **Female versus male** | 0.147682260 | 0.35601541 | 0.6792 |
| **BMI** | 0.027604017 | 0.02367240 | 0.2465 |
| **Current smoker** | 0.439093095 | 0.27280847 | 0.1108 |

1. Log(methylated INS DNA) as outcome

| **Parameter** | **Estimate** | **Standard Error** | **P value** |
| --- | --- | --- | --- |
| **Intercept** | 0.4254657223 | 0.62855632 | 0.5001 |
| **Group: HIV+CD4>350 vs HIV-** | -.4151079150 | 0.24470992 | 0.0931 |
| **Group: HIV+CD4<350 vs HIV-** | -.5945517584 | 0.30606058 | 0.0550 |
| **Group: HIV+ART+ vs HIV-** | 0.8384255085 | 0.32798363 | 0.0122 |
| **Age** | -0.012744895 | 0.01014046 | 0.2119 |
| **Black versus non-black** | 0.564165975 | 0.21186357 | 0.0091 |
| **Female versus male** | -0.382554803 | 0.25827376 | 0.1419 |
| **BMI** | 0.036501287 | 0.01717330 | 0.0361 |
| **Current smoker** | 0.462797301 | 0.19791073 | 0.0215 |
